# From apprehension to advocacy: a qualitative study of undergraduate nursing student experience in clinical placement in residential aged care

**DOI:** 10.1186/s12912-018-0277-z

**Published:** 2018-03-01

**Authors:** Heather Moquin, Cydnee Seneviratne, Lorraine Venturato

**Affiliations:** Faculty of Nursing, University of Calgary, 2500 University Dr NW, Calgary, AB T2N 1N4 Canada

**Keywords:** Nursing students, Nursing education, Action research, Residential aged care facilities

## Abstract

**Background:**

Undergraduate nursing placement in aged care is forecast to grow in importance with the increasing aging population, and to help to reverse trends in student lack of interest in gerontology careers. However, there is a need to better understand undergraduate nursing students’ experiences on placement with older adults, as well as key features of quality learning within residential aged care. The aim of this study was to explore how nursing students understand learning within residential aged care.

**Methods:**

This qualitative study used a participatory action research approach, and this paper reports on the thematic analysis of data from one cycle of undergraduate nursing placement in a Canadian residential aged care setting, with two groups of 7–8 students and two university instructors. Staff and residents at the research site were also included. Researchers interviewed both groups of students prior to and after placement. Instructors, staff and residents were interviewed post placement.

**Results:**

Students commenced placement full of apprehension, and progressed in their learning by taking initiative and through self-directed learning pathways. Engagement with residents was key to student learning on person-centred care and increased understanding of older adults. Students faced challenges to their learning through limited exposure to professional nursing roles and healthcare aide/student relationship issues. By placement end, students had gained unique insights on resident care and began to step into advocacy roles.

**Conclusions:**

In learning on placement within residential aged care, students moved from feelings of apprehension to taking on advocacy roles for residents. Better formalizing routes for students to feedback their unique understandings on resident care could ensure their contributions are better integrated and not lost when placements end.

## Background

### Creating an age friendly workforce – a difficult but necessary endeavour

“Because most patients in almost all settings are older, there is little place for the nurse who does not wish to work with older adults or who lacks the knowledge to provide this care” ([1], p. 24). Nurses must therefore emerge from their preparatory education ready and willing to provide the specific nursing care this population requires [2]. However, there is a general lack of interest among nursing students in gerontology careers [1, 3, 4]. This has been linked to persistent broad-scale ageism within society [1, 4, 5]; occurrences of negative stereotypes towards working with older adults within the nursing profession [6]; drops in students’ interests in gerontology careers as they progress through their studies [6, 7]; and a dominance of a cure versus care focus within health care [8]. Nursing schools have also been described as providing insufficient education on care of older adults [9].

This is further complicated by challenges in developing quality clinical learning environments for nursing students to learn and work with an older population. A growing shortage of nurses in Canada [10], means that nursing schools are under increased pressure to develop innovative educational programs and augment numbers of nursing professionals prepared for delivering on future healthcare needs [2, 11, 12]. Such interlinked understandings help to clarify why the development of an age-friendly nursing workforce is certainly not a straight-forward task.

Despite its limited use thus far, residential aged care (RAC) is forecast to grow in importance as a site for undergraduate nursing education [2]. Placement within RAC settings, though traditionally used early in nursing programs for education on basic nurse competencies, offers potential for advanced knowledge and practice [2]. Cartwright ([13], p. 243) notes that: "Community-based long-term care provides abundant opportunities for transformative learning and practice in areas that are core to 21st century nursing: managing chronic illness and palliative care in ways that are patient-centred and evidence-based, working in interdisciplinary teams, supervising unlicensed caregivers, and developing systems for ongoing quality improvement."

"Therefore, RAC is a key setting to kindle student interest in the care of older adults, while developing essential and advanced proficiencies in contemporary nurse practice."

To ensure placements in aged care settings contribute to the preparation of an age-friendly nursing workforce, the diverse influences on undergraduate student perceptions of practicum learning in aged care and with older adults must be explored. Evidence already suggests that students tend to find these placements “unsatisfactory and/or unsettling” and experience a sense of powerlessness on placement, reinforced by financial constraints, an unaccommodating learning/working environment, and a lack of readiness to encounter and care for ageing bodies ([14], p. 14). A further challenge with gerontological placements is that they are often located within impoverished care environments [7, 8].

Clinical placement is a key contributor to cementing student attitudes [8], and much effort is being made to counter these negative trends by designing placements in aged care that positively shape student perceptions. These efforts range from ensuring placements involve interaction with older adults in diverse settings [4], integrating creative and reflective pedagogies for shifting perceptions [15], and enriching placement environments [7, 8]. Within this body of work, it is important to maintain distinction between students’ perceptions of older adults and their perceptions of gerontological careers [5]. Within the context of a clinical placement with older adults, the aim of this study was to explore how nursing students understand learning within residential aged care.

## Methods

### Design

This paper reports on one 13-week undergraduate placement cycle that occurred in the summer of 2016. The study followed a participatory action research (PAR) design, involving cyclic, iterative processes for investigation, action, reflection and collaboration in the inquiry [16]. Action research methodologies are established approaches for practitioner research [17] and are particularly useful for nurse researchers in centring the research endeavour within the context being studied and involving research participants as partners in the inquiry [18]. A thematic qualitative approach was used for data analysis, a method often used within action research for uncovering meaning [17].

### Development of a positive person-centred placement model

The first stage of this research involved the collaborative and ongoing development of a person-centred placement model aimed at enhancing the interests of undergraduate nursing students in working with older adults. In the spring of 2016, a working group consisting of university educators and researchers, site staff, and residents met in seven 1-h sessions to begin to co-develop the placement model. The working group developed and planned a person-centred placement model for the upcoming student placement, incorporating elements such as the concepts of person-centred care and intergenerational learning; student and resident orientations; communication of student skills and developing capabilities; and roles and expectations of residents, staff, and students.

### Setting

Two groups of undergraduate nursing students, with 1 instructor respectively, were placed at two supportive living facilities owned and operated by the same non-profit provider and co-located on the same campus. The 13-week practicum cycle (249 total hours) occurred in the summer of 2016.

#### Facility 1: positive person-centred placement model

At the intervention site, eight students and residents were matched by the working group and structured time was accommodated in the student schedule. Students spent 1.5 days per week at the sites and worked with healthcare aides (HCAs), particularly during morning care routines. Students also worked one-to-one with residents on a care plan assignment.

#### Facility 2: traditional clinical placement model

The second site used a traditional clinical placement model. Here, seven students chose which residents to work with and their time with residents was less structured. Like Facility 1, students spent 1.5 days per week at this site, worked with HCAs, and worked one-to-one with residents on a care plan assignment.

While there were some variances between the placement models at site 1 and 2 in relation to orientation to site, intentionality and focus in the student–resident interactions, in general the placement model at the intervention site was not fully implemented and there were minimal differences between groups. Therefore findings for both groups are presented together to further understanding of student learning within RAC.

### Participants

Qualitative data was collected with both groups of students (*n* = 15) and instructors (*n* = 2), and with site staff (*n* = 8) and residents (*n* = 5) involved at the intervention site. Instructors in this study were experienced RNs employed by the university, on a contractual basis, to accompany and facilitate undergraduate student learning during clinical placement. Student participants were second year, first term undergraduate students undertaking their first clinically focused placement. Staff included were HCAs, licensed practice nurses (LPNs) and site management (RNs and allied health personnel).

### Data collection

Focus groups were held with students pre- and post-placement, and with residents and staff post placement. Individual interviews were held with instructors and site management post-placement. Data collection was conducted by two members of the research team and sessions took one hour. Interviews and focus groups were conducted in a meeting room at the site or university. Interviews and focus group questions explored participants’ experiences during the placement, and learning facilitators and challenges. Questions were framed temporally (see Table 1 in Appendix for a list of questions by participant group). Focus groups and interviews were all digitally recorded and transcribed verbatim.

### Data analysis

Data was analyzed thematically [17]. Transcripts were read and analyzed by two of the authors. Initial readings and analysis resulted in general theming of the data. A series of meetings between the authors allowed for agreement on a set of broad themes under which sub-categories were placed. Meetings between the authors on the initial draft prompted a temporal frame as a useful lens for analysis. Subheadings were created to structure and frame the writing so that it flowed temporally from anticipation and beginning placement to completion and final reflection. The authors included further data related to ‘process’ and redrafted the writing until coming to an agreement on the findings.

## Results

This study found that undergraduate nursing students began their placements in RAC with a range of apprehensions and expectations. On placement, encountering challenges in working with HCAs and with a lack of professional nursing roles, students sought out mentorship and learning opportunities. Pairings with residents on placement helped students gain appreciation for older adults and learn person-centred care. Over time and with encouragement, students developed initiative and capacity so that by the end of placement they more actively created their own learning opportunities and stepped into advocacy roles for residents.

### Beginning placement: Concerns and expectations

Both groups of students felt hesitant and had concerns prior to placement. One student explained, *“I was just nervous in general…I didn’t really know what we would be expected to do”* [N-S-P7]. With this being their first practicum involving care provision, students were acutely aware of their possible limitations: *“I was…concerned about not being ready or not knowing my skill set”* [P-S-P5]. Students felt uncertain about what they would be doing and on their preparedness for the placement. One student explained, *“I didn’t know how I was going to handle peri care…I didn’t know how I was going to react to that”* [P-S-P6].

As learners in the environment, students worried they might get in the way, upset routines, or become a bother to staff or residents. Students explained that they did not want to be ‘useless’ and there was worry about how staff might perceive them: *“We really haven’t had a placement with a nurse yet so…you just don’t want to be like the annoying student that just annoys the nurses and makes their job difficult”* [P-S-P4].

Students also worried about their abilities in care provision and interacting with residents. One student explained, *“some people really hate their hair being brushed in a certain way and you are not going to figure it until you do it…that was where I was like a little nervous”* [N-S-P2]. Concerns related to care of residents were often associated with limited previous experience with older adults and with dementia. Where students had past experiences with LTC, there was apprehension on whether the care environment would be positive or not, and the emotional and practical implications that might have: *“My great aunt was in a residence so I guess it was more the environment, if it was going to be a happy environment”* [P-S-P6].

It was clear that students wanted to gain skills in care delivery and learn person-centred care from their placements. One group explained that they would like to learn everything *“hands-on”* [N-S-P1] and *“everything that a healthcare aide does basically, [and] a little bit of what an LPN does”* [N-S-P5]. Another student felt that learning in this environment was about getting comfortable with the tasks learnt at the university: *“[It’s] a comfort level…just doing certain tasks like the morning care…feeling … comfortable in doing it by myself”* [N-S-P3]. Learning hands-on skills extended for some to include aspects of person-centred care: *“I don’t necessarily mean hands on skills in terms of like starting IVs and…catheters, but …I have learned about communicating with older adults with dementia”* [N-S-P3]. Many students discussed how they hoped to learn aspects of person-centred care on placement through communication with residents and their families, enriching quality of life, and delivery of care beyond a task focus. Students wanted to learn person-centred care alongside *“how you maintain like the medical side of things…how patient care is delivered…while also keeping it personal and individual and how those two kind of marry”* [P-S-P5].

### On placement: Seeking mentorship and learning progression

Students worked with HCAs for morning care on these placements, which allowed them to build their skills in the delivery of personal care of residents. By the end of placement, students explained that working with HCAs allowed them to learn to recognize differences in quality of care that were key to their understandings of person-centred care:

*If you have a really good [HCA] you work with, you can see how it really sets [residents] up for a really good day and makes things a lot better so you can really see how that care impacts the residents* [P-S-P2].

Role hierarchies and lack of role clarity caused awkwardness and general anxiety, and meant that both students and HCAs were often unsure on the best ways to work together. As one resident put it, *“already the students are more educated than the [HCAs] and the [HCAs] have more experience so I’m sure it was awkward”* [P-R-P1]. HCAs were unsure of the tasks and skills students could do and were reluctant to provide students with learning opportunities. This was frustrating for students: *“It’s not like we lack motivation…someone needs to tell us what we are supposed to do…if there is zero instruction there, it can be a little bit uncomfortable because you feel like you are in their way”* [N-S-P3]. One of the instructors explained that this largely had to do with teaching being outside HCA scope:

*[HCAs are] not equipped to take students…and you can’t fault them either because within their scope it’s…task-orientated nursing. So for them to take on a BN student…it can be challenging because they’re not sure what level our students are at, what they can do and can’t do* [N-I-P1].

Even when provided with a list of student skills, HCAs were still reluctant to undertake mentoring. Students felt they were chasing HCAs and felt abandoned when HCAs were busy in their routines and did not have time or skillset to consistently integrate the student into potential learning opportunities. As one student explained, HCAs and students danced around each other: *“We were asking: what would you like us to do? And they are like, what can you do? What have you been doing? They don't have time to like hear that at 7 in the morning”* [N-S-P1].

It was crucial that students developed confidence in their abilities and learned to take initiative in their learning. Confidence and initiative took time to develop, but both groups of students became more confident over the period of the placement and began to have greater autonomy and independence in their own learning and in the delivery of care: *“We just over the time got more comfortable working on our own”* [N-S-P1]. Over time students became more at ease actively seeking out opportunities: *“It will take a few weeks for you to figure out what you want to do and what you haven’t learned yet…to ask those specific questions too”* [P-S-P1]. In some cases, students needed to be more direct about gaining opportunities they felt they needed: *“I spent a lot of time…observing at first…it was until the end that I realized like if I want to make something happen…I just have to force my way in there”* [P-S-P2]. Actively instructing students to take initiative helped students with this learning progression: *“[The instructor] prompted us…next day, ‘I want you to take control’ and we were like ‘okay,’ so we did”* [N-S-P4]. Where staff-student pairings were consistent, greater time on placement also helped staff become more familiar with student skillsets, which facilitated student learning: *“[We] got to know the staff as well, and they became comfortable working with us and understanding what our abilities were, so I found that helped also”* [N-S-P5]. Not setting up expectations that site staff would seamlessly provide mentorship seemed to also help bolster students to build their own capacity to seek out learning opportunities: *“I feel like it was kind of our job to make the learning opportunities that we wanted”* [N-S-P1]. It was up to the instructors and the students to maintain the educational focus and ensure learning took place. As one student explained, it was necessary for students *“to advocate for yourself [and] find things when you can”* [P-S-P4].

An important aspect to students being able to take initiative on placement was conceptualizing learning as self-directed. Students worked with their instructors to access the learning opportunities they felt they needed for their RN professional development. As one instructor explained, *“because everybody’s learning needs are different…their skillsets were theirs to try and identify…I tried to help them find those opportunities”* [P-I-P2]. There was an element of chance with this, where students needed to be ready and willing to grab any learning experiences that came their way: *“In the morning, if something came up we were like ‘yes’! I would absolutely jump in there and do it, if they allowed us to. That was the best way in our unit to get those opportunities.”* [N-S-P5].

Instructors also encouraged students to create the learning opportunities they required, which sometimes meant resituating repetitive task-based activities for aspects relevant for their education. For example, while serving breakfast to residents many students spent time conversing and getting to know residents, which strengthened their understanding of person-centred care. Instructors took care to build student capacity and agency over the placement period so the process was progressive. For example, students were encouraged to begin working in pairs and not *“break off from your pairs until after mid-term and start taking more initiative and more responsibility to your workload”* [N-I-P1].

Students felt that RN and LPN job shadowing opportunities would have been a natural learning progression while on placement:

*I think it would be beneficial to have like a progression. Like start working with the [HCAs], learn what they do, learn their role and then maybe move on to the LPNs and shadow them and see what they do and move up to the RNs and see what they do in this facility* [N-S-P4].

Job shadowing was discussed as a way for students to learn about the more managerial aspects to nursing within residential aged care:

*I think I could have really benefited from some more like LPN and RN time…we’ve been learning…how like RNs are like case managers and all these different roles that they could have taken, so I would’ve benefited from seeing how an LPN organizes their day and leads the team* [P-S-P8].

Though discussed as key opportunities for students, job shadowing of LPNs or RNs occurred very rarely at either placement site. As one student explained jokingly, *“we don’t get to hang out with the RNs much. They are like the cool kids”* [N-S-P5].

Limited RNs employed within these settings was an obvious barrier to job shadowing; however, a predominant view of nursing as ‘hands-on’ seemed to also contribute to this missed opportunity. One RN staff member explained, for example, *“sit[ting] in the office all day and push[ing] paper and call[ing] families on the phone…would not have given that student anything I don’t think”* [P-St-P1]. Despite acknowledgements that nursing within RAC has become more managerial, a preference to see, do and learn nursing as ‘hands-on’ was a predominant theme. These views seemed to block opportunities for understanding the role of the RN in LTC and learning progression beyond basic care.

### On placement: Engagement with residents and building relationships

Interactions with residents allowed for positive outcomes in student learning. Students at both sites worked with residents on a one-to-one basis to complete a care plan assignment. Relationship building between students and residents also occurred through more generalized time together during placement, from students assisting with personal care of residents to spending time together in everyday activities such as mealtimes.

Spending time together enabled students and residents to build positive relationships. Residents discussed how they really enjoyed spending time with the students, students felt their time with residents was a consistent highlight of their placements, and site staff were grateful for the extra quality time students could provide to residents. While residents understood that these relationships may not last beyond placement, that the students were *“here for a purpose”* with a *“full schedule”* [P-R-P1], this did not detract from the positive bonds built while the students were there. It was clear that the residents missed the students after the placement ended and were very much looking forward to the next placement and having students on site again.

Lack of experience with older adults prior to placement had contributed to students’ early apprehensions: *“My concerns were just like lack of practice, skills and just lack of exposure to older adults”* [P-S-P2]. Reflecting after placement, students confirmed that their perceptions of older adults had changed: *“I didn't think that I would enjoy working with older adults as much and I really did enjoy working with them”* [P-S-P2]. An increased understanding of older adults came from having the chance to spend extended time with a resident and sharing life stories and lessons: *“The person I worked with she…lived her life and it didn't matter what happened; she looked forward, she didn't dwell on what was happening to her…her outlook was amazing and you learn a lot from them I think*” [P-S-P1]. Students gained appreciation both for the residents they paired with and for older adults more generally: “*I would say the one word: underestimate. Do not underestimate them*” [P-S-P6].

Greater understanding of older adults was often linked to recognizing the commonality of human experience students shared with older adults. As one student explained, *“they are just as fragile as us”* [N-S-P7]. Student learning also extended to recognizing unique attributes of older adults:

*Of course you treat older adults like you would treat anybody else…but then also seeing like why that can be challenging. Like all these people are dependent on other people and of course they don’t like to be…there are just challenges that I never realized* [N-S-P1].

Engagement with residents also allowed students to refine and practice their understandings of person-centred care. Students emphasized how greater familiarity with residents through *“trying to get to know the patient…personally and not just…from their charts or records or whatever you see in their reports”* [N-S-P6] allowed the care provider to gain a sense of personality and behavioural norms for each individual resident. This familiarity was seen by the students as a basis to be able to deliver care that is person-centred: *“Understanding who the client is, and then you can see fluctuations in that and you can do…your care based on that… baseline”* [P-S-P7]. Person-centred care was described by the students both as integration of life histories into care tasks, as well as an extra aspect to care routines: *“just having the time to take that extra two minutes…to sit with them and listen…just that extra…communication piece”* [N-S-P6]. Prioritizing unique engagement with each resident also meant learning to dismiss broad stereotypes: *“Not making assumptions like you are old you can't do this…just ask them like how would you like to do this or what works best for you…because they know their body best and they know what works”* [P-S-P3]. Students recognized that person-centred care can be more of a challenge for staff due to time and budget constraints, but felt it was crucial for sites to deliver care that is person-centred: *“Nurses are busy and it is hard to take the extra time…just taking the time to actually wait and listen…that patient space is really important…to find out the whole story”* [N-S-P4].

Engagement with residents with or without dementia was differentiated, but both opportunities were appreciated. Because learning was self-directed, students worked with their instructors to build in rotations to different units that they felt worked best for their learning. However, placement in supportive living did provide students with less experience with dementia than they would have liked:

*It sounds like most of the other [students] were like in [a] long term care home so a higher level of care…that is something that still kind of makes me a little uneasy is I don’t feel like I’m that comfortable in dealing with people let’s say with really, really high levels of dementia* [P-S-P2].

For those who did have those opportunities, working with residents in a memory care unit allowed students to learn specific strategies for working with people with dementia. One student explained that getting to know and observe one resident over time allowed her to recognize changes in eating patterns even when communication was difficult:

*One of the really good eaters if they are not eating breakfast…Okay, well that’s not how this person normally is. So, even though he can’t communicate with me if he is not feeling well, just because I know him as an individual then I am able to assist him better* [N-S-P4].

Students were able to build different types of relationships than those staff have with residents. As one student explained, *“there is a different feel for nursing students; like our presence here compared to the people who work here. They kind of treat us differently, well, I would say nicer”* [N-S-P1]. These different types of relationships allowed students to gain insights on residents beyond what staff know:

*For the care plan [assignment] I got to know like her background and where she was coming from…she loves to be alone on her room but with me I got to know like the reason behind that, which I don't think the [HCAs] had time to know cause they were always busy, or the LPNs…she wouldn't really trust…the workers there but, me like I am the outlier, I am the student so she was more open to me…I got to know the reason why she acts the way she acts. That's something they were missing* [N-S-P6].

This different element to student-resident interactions was attributed to the fact that these exchanges were framed as educational and not carrying the same “real-life” weight as staff-resident interactions: *“[Residents] were open to helping you and answering your questions, doing assessments and like kind of letting you role play with them…because our assessments don’t really go into their chart so there’s no threat there”* [P-S-P3].

### Reflections on placement: Finding a role in RAC

A lack of insight into the RN role, student preference for hands-on nursing, and systemic shifts in the role of the RN away from direct care delivery all contributed to dissuading students from envisioning themselves working in these settings in the future. When asked if they would work in aged care, most students responded that without spending enough time with practice RNs, they were still unsure on the RN role and scope of practice: *“I really want to work on long-term care; I thought it would be great but then having worked or done this placement I don't really know how the RN fits in”* [P-S-P7]. Despite spending very little time with RNs, students recognized the more administrative nature to the RN role and expressed a preference for nursing that was more hands-on with greater resident contact:

*I don’t think I’ve seen enough of the RN to be like ok this is what I want to do…I’m definitely more hands on so I don’t want to like sit in the office and do like documenting and like stuff like that so for that reason I would probably…not do long term care* [P-S-P6].

Students recognized that systemic pressures were shifting the RN role away from hands-on nursing, which also contributed to the predominant choice of not wanting to work in RAC: *“[RNs at the bedside] would be something I would be interested in doing…because [older adults] are definitely like an interesting dynamic and like a great population but it is unfortunate the system doesn't support it”* [P-S-P8].

Despite not envisioning themselves working in RAC, students recognized that knowledge specific to the care of older adults is very much needed beyond this setting and envisioned themselves working with older adults in other settings. As one student summarized, *“no matter which setting you are working in, in health care you will be dealing with older adults and you can learn so much from that experience with them”* [P-S-P1].

Where students learned greater appreciation for older adults and person-centred care within these placements, some felt this would not have been as easily learned in acute settings. It was seen as beneficial to learn these skills here in a *“safe environment where you can constantly be interacting with people, where it is not changing everyday like in an acute setting”* [P-S-P1]. These aptitudes were seen by students as transferable to other settings and applicable to their future provision of care to older adults:

*Learning how to build the relationships with older adults…I hopefully will end up working in a hospital setting…so you know where they are coming from and then when they leave the hospital where they are going back to…how can I best set them up for success as they go back to the long-term care setting* [P-S-P4].

In the face of systemic pressures impacting aged care settings, students took steps towards placing themselves in advocacy roles for residents. With current restraints on staff time and quality of care, students felt residents needed greater advocacy: *“They just need someone…who could advocate for them…because everyone else is so busy running around just trying to help them with ...daily activities”* [P-S-P3]. In taking initiative, students tried to help provide quality resident care:

*The lady who had an edema in her leg, and the [HCA] was putting on her pressure socks…she was quite rushing [*sic*]… ‘why don't you just go to the next client while I just take my time with this one?’ Because…I [can take] my time to put on her pressure socks. Less pain* [N-S-P6].

## Discussion

This study aimed to explore how undergraduate nursing students understand learning on placement in RAC. It was found that students began their placements with various apprehensions and expectations before taking more initiative and seeking out learning and mentorship opportunities. Learning on placement was affected by challenges in students’ relationships with HCAs and little exposure to professional nursing roles, impacted by a prevailing view of nursing as hands-on. Students spent extra time with residents and began to step into advocacy roles by placement end. Pairings with residents contributed to students gaining greater appreciation for older adults and learning person-centred care.

### Theme 1: Moving on from initial apprehensions by taking initiative

Students began their placements experiencing a mixture of different apprehensions. Anxiety when first undergoing placement is recognized across the literature as students encounter “the culture and ethos of nursing with its complexities and challenges…often for the first time [which]…can be both terrific and terrifying” [15, p. 31]. In this study, along with general anxieties about the newness of the setting and concerns over their own readiness and skillsets, students explained that they worried about how to most usefully interact with staff and residents, while simultaneously learning and gaining tangible skills. Brown et al. [8] developed a temporal model of student placement with a progressive shift of student foci—from self, course, professional care, to patient as person—depending on the support provided by their mentorship and learning environment. The first stage of this model aligns with our findings on student apprehensions, as Brown et al. [8] indicated that students’ experience anxiety and insecurity as outsiders within their first placement environment, looking to fit in while also accomplishing their learning outcomes.

As outsiders, students’ work with HCAs was consistently awkward and challenging. This was attributed to role hierarchies, uncertainties around student skill-sets, mentoring as beyond HCA scope, and concerns over the best ways to work together. In a comprehensive study investigating capacity of RAC settings to support student placement, students reported encountering similar challenges, including few learning opportunities and being asked to observe rather than engage in care provision [19]. Such challenges are brought into immediate focus with the study by Robinson et al. [19] that detailed how staff working in RAC consistently *live on the edge* in their roles, which provides little-to-no excess capacity for contributing to student learning on placement. Though research on HCA-student interactions in aged care placement is scarce, a study by Annear et al. [20] developed an educational resource – a Carer Assessment and Reporting Guide – to better prepare HCAs and undergraduate nursing students to learn and relate interprofessionally. Our findings confirm the need for further research in this area to better understand the challenges and to develop further strategies for improving the relationships between HCAs and students within RAC placements.

Students reported an element of *chance* to their learning opportunities, a recognized element of the clinical experience: “education by random opportunity” ([21], p. 170). To move beyond chance, it was up to students to progressively take initiative in their relationships and in the setting to ensure they accessed the learning opportunities they wanted and required. Taking initiative is recognized as a key element contributing to a positive RAC placement experience for students [22] as it complements development of self-reliance [8] and is an important component to active involvement and learner responsibility on clinical placement [23]. In our study, support and encouragement for self-directed learning from instructors was important in allowing students to move beyond their initial anxieties to actively seek out and accomplish their learning goals.

### Theme 2: Learning progression through job shadowing

Along with fewer numbers of RNs working in RAC—a decline recognized even a decade ago [19]—a predominant view of nursing as hands-on care seemed to limit the opportunity for students to have exposure to RN/LPN roles. With limited insight into RN and LPN roles, students felt unsure about their future role, and failed to see value in professional practice in this setting. Abbey et al. [14] similarly found that limited exposure to RNs left students with little to no understanding of the complex responsibilities involved with the role and the professional supports available. Students in our study consistently described a lack of RN/LPN exposure as a missed learning opportunity.

Despite having little exposure to RNs, students were aware that the RN role in this setting has shifted away from hands-on care towards a more administrative focus. The literature provides some evidence for why students may hold preferences for hands-on nursing. Ironside et al. ([24], p. 188) explained that students undertaking clinical education tend to be very focused on task completion to the point where “doing more tasks” is equated with "promot[ing] learning and ensur[ing] success in [their] clinical course", despite this potentially being at odds with comprehensive practice learning. Further, Abbey et al. [14] found that students conceptualize ‘real nursing’ as technical skills – a message reinforced through popular culture – and surmised that a ‘normalization movement’ in RAC, or purposely enhancing the domesticity of these settings, can mask the clinical content and reinforce student perceptions that these settings are de-medicalized requiring less technical skills. Further, undergraduate nursing students can feel ill-prepared and intimidated by the managerial role of RNs working in nursing homes [25]. These three elements of student focus within clinical education—an overemphasis on task completion, an underappreciation of technical content within aged care and a lack of preparation for administration—helps to provide greater context to a preference for hands-on nursing evident within our study.

Comparisons of hands-on nursing versus ‘sitting in the office’ within the data highlight how hands-on care is also seen as contact with residents and tied closely to nursing professional identity. With little exposure to RNs working in the setting and with an understanding that RNs work in more managerial positions, students explained that they wanted to work in future positions that involved contact with residents – and felt that the RN role in RAC would not provide them with that. Aged care nurses themselves feel less satisfaction with their roles, due to greater responsibilities and lack of role clarity, and this has been linked to a limited capacity of some RAC sites to support clinical placement [19]. Care must be taken to ensure students have positive experiences in aged care nursing, this ultimately informs their career decisions [19, 26]. With the real challenges facing aged care nursing as a profession, support for student learning requires exposure to professional role models who can highlight and communicate positive aspects of aged care nursing [1, 14].

Greater exposure to positive professional nursing roles could help promote the reality of nursing as a profession involving a broader spectrum of knowledge and services, including administration and case coordination. Abbey et al. ([14], p. 17–18) explain that “exposure to, the decision making, care planning and assessment procedures undertaken by registered nurses in aged care. . . may help to illustrate that the role is more demanding and better supported than the students are easily able to see.” Overtly designing placements that go beyond basic competencies could also positively influence student perceptions of working in RAC [14] and could help dispel perceptions that aged care nursing requires lesser skillsets and know-how than nursing in acute care settings [4].

### Theme 3: Gaining appreciation for older adults and moving into advocacy roles

During placement, students engaged one-to-one, spent extra time generally with residents and formed relationships with older adults living in the aged care setting. These opportunities helped students develop their understandings of person-centred care and gain greater appreciation for older adults. Aud et al. [4] indicated that students gain greater respect for older adults and learn to dispel stereotypes through spending time, and having the chance to interact and build relationships. Students also gained appreciation for older adults through recognizing their shared experiences, as well as the unique aspects of the lives of older adults. Importantly, students come to understand older patients as ‘people’ when they are able to relate the experiences of older people to their own lives [8]. Further, Elliot et al. [27] identified that residents and family members felt student placement in RAC resulted in a wide array of benefits, including increased social interaction. Indeed, social interaction with students was reported by residents in our study as rewarding and enjoyable.

Spending time with residents facilitated unique insights for students, who used these to step into resident advocacy roles. At both sites, students worked to integrate their unique insights into care delivery through using the extra time they had with residents to build personal relationships, and deliver person-centred care and individually targeted interventions for residents. Feeding back these insights at the organisational level was more problematic. As outsiders, students have little power to enact change in resident care or access formalised processes within the organisation. Elliot et al. [27] discussed student placement in RAC as a unique way to increase capacity in an over-strained sector. That study reported that students on placement improved quality of care and oversight, and offered unique supports to the sector, shifting the focus from managing workforce issues to learning – though the ethical implications to accommodating the student role in care delivery are underscored.

Where students in our study described being able to build different types of relationships with residents than staff, the potential of student presence on placement supporting person-centred care and resident advocacy is highlighted. Intergenerational service learning initiatives align learning objectives with community-identified priorities [28] and have become a “global phenomenon” over the past decade in clinical nursing education, particularly for placement in community settings ([29], p. 378). With their ability to directly incorporate student advocacy for patients into learning (e.g. [30–32]), integrating intergenerational service learning approaches into aged care placement may better support and utilize the extra role of students on placement. Supporting a more active role for students and formalizing ways for student perspectives to be fed back could help to ensure their unique contributions are better integrated into ongoing site operations and not lost when placements end.

### Limitations & suggestions for future research

This study focused on two undergraduate student groups from the same university within the supportive living settings of one RAC organization, therefore the generalizability of the findings is limited. However, the study provides insights on student learning trajectories, integration of staff mentorship to support student learning and the benefits of student-resident engagement opportunities within placements in RAC. There is a need for further investigation into the potential benefits of communicating self-directed learning pathways to students, methods for better integrating professional nursing roles into student learning in RAC and formalizing feedback of unique contributions gained through student-resident engagement opportunities.

## Conclusion

This study found that taking initiative was important for undergraduate nursing students to move from initial apprehensions to create their own learning opportunities and begin to step into resident advocacy roles while on placement in RAC. Similar student trajectories on placement within RAC are reported in the literature, and methods to enrich mentorship and learning environment are highlighted [8, 20, 22, 26]. Conceptualizing their learning as self-directed was key to students undertaking learning progression within our study. It would be ideal to communicate self-directed learning pathways to students undergoing their first placements in RAC, not to offer prescriptive templates to follow, but to better prepare novice students, set expectations, and optimize learning on placement.

Placements in RAC can better support positive learning progression pathways through including RN and LPN job shadowing experiences for students. However, where there are consistently low numbers of senior staff in RAC to contribute to student learning, there must also be real work accomplished on affirming and communicating the value of care aide competencies to students training to be RNs and easing the working of this key relationship within placement. Shaping learning progression pathways within aged care placements as positive with clear guidelines on how sites can contribute to student mentorship could provide greater opportunity for students to gain a more fulsome understanding of the nursing profession within RAC. This may help set the groundwork for students to better picture themselves working in these settings in the future.

Including student-resident engagement opportunities on placement was found to help students build their understanding of older adults and learn aspects of person-centred care. Where students had extra time to spend with residents and as these interactions were framed educationally, students described building more personal relationships with residents than staff and gaining unique insights into the lives of residents. Better formalizing and supporting this extra time and attention students spend with residents while on placement could facilitate the unique contributions of students into care delivery.

## Appendix

**Table 1:** Interview & Focus Group Questions by Participant Group

Participant Group	Interview/Focus Group Questions	
	pre	post
Students	At end of placement, what do you hope to have learned? How have your courses prepared you for this placement?	Tell us about your experience learning on this placement. (Learning from staff, residents, instructors, highlights, challenges) How has your placement experience shaped your perceptions of older adults/work with older adults?
	post	
Staff/Residents/Instructors	Tell us about your experience with this placement. (highlights, challenges)	

## Abbreviations

HCA: Healthcare aide
LPN: Licensed practical nurse
LTC: Long-term care
RN: Registered nurse

## References

[1] Cozort R (2008). Student nurses' attitudes regarding older adults: strategies for fostering improvement through academia. Teach Learn Nurs.

[2] Melillo KD, Abdallah L, Dodge L, Dowling JS, Prendergast N, Rathbone A, Remington R, Shellman J, Thornton C (2014). Developing a dedicated education unit in long-term care: a pilot project. Geriatr Nurs.

[3] Burbank PM, Dowling-Castronovo A, Crowther MR, Capezuti EA (2006). Improving knowledge and attitudes toward older adults through innovative educational strategies. J Prof Nurs.

[4] Aud MA, Bostick JE, Marek KD, McDaniel RW (2006). Introducing baccalaureate student nurses to gerontological nursing. J Prof Nurs.

[5] Gould ON, MacLennan A, Dupuis-Blanchard S (2012). Career preferences of nursing students. Can J Aging.

[6] Higgins I, Van Der Riet P, Slater L, Peek C (2007). The negative attitudes of nurses towards older patients in the acute hospital setting: a qualitative descriptive study. Contemp Nurse.

[7] Brown J, Nolan M, Davies S, Nolan K, Keady J (2008). Transforming students’ views of gerontological nursing: Realising the potential of ‘enriched’ environments of learning and care: a multi-method longitudinal study. Int J Nurs Stud.

[8] Brown J, Nolan M, Davies S (2008). Bringing caring and competence into focus in gerontological nursing: a longitudinal, multi-method study. Int J Nurs Stud.

[9] O’Lynn C (2013). Comparison between the Portland model dedicated education unit in acute care and long-term care settings in meeting medical-surgical nursing course outcomes: a pilot study. Geriatr Nurs.

[10] Canadian Nurses Association. Tested solutions for eliminating Canada’s registered nurse shortage. 2009. https://cna-aiic.ca/~/media/cna/page-content/pdf-en/rn_highlights_e.pdf. Accessed 23 March 2017.

[11] Canadian Nurses Association. Policy Brief #2: Meeting future healthcare needs through innovations in nursing education. 2013. https://www.cna-aiic.ca/~/media/cna/files/en/hhr_policy_brief2_e.pdf?la=en. Accessed 1 April 2016.

[12] Barnett T, Cross M, Jacob E, Shahwan-Akl L, Welch A, Caldwell A, Berry R (2008). Building capacity for the clinical placement of nursing students. Collegian.

[13] Cartwright JC (2010). Opportunities for practice and educational transformations through unlikely partnerships. J Nurs Educ.

[14] Abbey J, Abbey B, Bridges P, Elder R, Lemcke P, Liddle J, Thornton R (2006). Clinical placements in residential aged care facilities: the impact on nursing students’ perception of aged care and the effect on career plans. Aus J Adv Nurs.

[15] Brand G, McMurray A (2009). Reflection on photographs: exploring first-year nursing students’ perceptions of older adults. J Gerontol Nurs.

[16] McIntyre A (2008). Participatory action research.

[17] McAteer M (2013). Action research in education.

[18] Streubert HJ, Streubert HJ, Carpenter DR (2011). Action research method. Qualitative research in nursing: advancing the humanistic imperative.

[19] Robinson AL, Andrews-Hall S, Fassett M (2007). Living on the edge: issues that undermine the capacity of residential aged care providers to support student nurses on clinical placement. Aust Health Rev.

[20] Annear M, Lea E, Robinson A (2014). Are care workers appropriate mentors for nursing students? An action research study in residential aged care. BMC Nurs.

[21] Leflore JL, Anderson M, Michael JL, Engle WD, Anderson J (2007). Comparison of self-directed learning versus instructor-modeled learning during a simulated clinical experience. Simul Healthc.

[22] Lea E, Marlow A, Bramble M, Andrews S, Crisp E, Eccleston C, Mason R, Robinson A (2014). Learning opportunities in a residential aged care facility: the role of supported placements for first-year nursing students. J Nurs Educ.

[23] McIntosh A, Gidman J, Smith D (2013). Mentors’ perceptions and experiences of supporting student nurses in practice. Int J Nurs Pract.

[24] Ironside PM, McNelis AM, Ebright P (2014). Clinical education in nursing: rethinking learning in practice settings. Nurs Outlook.

[25] King BJ, Roberts TJ, Bowers BJ (2013). Nursing student attitudes toward and preferences for working with older adults. Gerontol Geriatr Educ.

[26] Lea E, Marlow A, Bramble M, Andrews S, Eccleston C, McInerney F, Robinson A (2015). Improving student nurses’ aged care understandings through a supported placement. Int Nurs Rev.

[27] Elliot KJ, Annear MJ, Bell EJ, Palmer AJ, Robinson AL (2014). Residents with mild cognitive decline and family members report health students ‘enhance capacity of care’ and bring ‘a new breath of life’ in two aged care facilities in Tasmania. Health Expect.

[28] Karasik RJ, Berke DL (2001). Classroom and community: experiential education in family studies and gerontology. J Teach Marriage Fam.

[29] Knecht JG, Fischer B (2015). Undergraduate nursing students’ experience of service-learning: a phenomenological study. J Nurs Educ.

[30] Bell ML, Buelow JR (2014). Teaching students to work with vulnerable populations through a patient advocacy course. Nurse Educ.

[31] Hamel PC (2001). Interdisciplinary perspectives, service learning, and advocacy: a nontraditional approach to geriatric rehabilitation. Top Geriatr Rehabil.

[32] Hermoso J, Rosen AL, Overly L, Tompkins CJ (2008). Increasing aging and advocacy competency. J Gerontol Soc Work.

